# Accumulation Profiles of Embryonic Salt-Soluble Proteins in Maize Hybrids and Parental Lines Indicate Matroclinous Inheritance: A Proteomic Analysis

**DOI:** 10.3389/fpls.2017.01824

**Published:** 2017-10-25

**Authors:** Fen Ning, Xiaolin Wu, Hang Zhang, Zhaokun Wu, Liangjie Niu, Hao Yang, Wei Wang

**Affiliations:** State Key Laboratory of Wheat and Maize Crop Science, Collaborative Innovation Center of Henan Grain Crops, College of Life Sciences, Henan Agricultural University, Zhengzhou, China

**Keywords:** 2-DE, proteomics, maize embryo, salt-soluble proteins, hybrids, heterosis, non-additive proteins, globulin

## Abstract

Maize is one of the most widely cultivated crops. It accumulates a large quantity of seed storage proteins, which are important for seed development and germination, and contribute to the nutritional quality of seeds. Based on solubility, the storage proteins are divided into albumins (water-soluble), globulins (salt-soluble), prolamins (alcohol-soluble), and glutelins (acid- or alkali-soluble). Maize hybrids are cultivated due to the superior performance of F_1_ hybrids than that of their parents, a phenomenon known as heterosis. However, the accumulation patterns of seed storage proteins in maize embryos between the hybrids and their parental inbred lines have not been compared. In the present study, two elite inbred lines of China, Zheng 58 and Chang 7-2, and their reciprocal hybrids (Zheng 58 × Chang 7-2 and Chang 7-2 × Zheng 58) were used to explore parental influences on the accumulation patterns of seed storage proteins in maize embryos. For this purpose, we focused on seed salt-soluble proteins (SSPs) in our experiments. The SSPs were selectively extracted from maize mature embryos after extensive removal of water-soluble albumin and separated using two-dimensional gel electrophoresis (2-DE), followed by mass spectrometry analysis. Our results indicated that the 2-DE SSP profiles of hybrids closely resembled those of their maternal parent rather than the paternal parent. In other words, 2-DE SSP profiles of Zheng 58 × Chang 7-2 were more similar those of Zheng 58 whereas such profiles of Chang 7-2 × Zheng 58 were more similar to those of Chang 7-2 although the 2-DE profiles of all four maize types were quite similar. In total, 12 relatively abundant SSPs spots representing five kinds of proteins were identified, of which nine protein spots displayed non-additive accumulation in at least one hybrid. This study provided additional data on dominance and partial dominance effects on maize hybrids embryos. Besides, earlier studies on accumulation profiles of globulin-1 (also known as vicilin), which is one of the most abundant globulins in maize embryos, also support the above results. This study would be helpful in revealing the mechanisms underlying SSPs accumulation patterns in the hybrids.

## Introduction

Rice, wheat and maize are the most important crops in the world, accounting for over 70% of the total cereal species production (FAO, http://faostat3.fao.org/home/E). About 10–12% of the dry mass of cereal grains is the seed storage proteins, which are a major source of protein nutrition for humans and animals (Shewry and Halford, 2002). The seed storage proteins are synthesized and accumulated as nutrient reserves of amino acids for seed germination and early seedling growth (Herman and Larkins, 1999). Besides, these proteins significantly influence the utilization of cereal grains in food processing (Mandal and Mandal, 2000).

Seed storage proteins are empirically classified according to their solubility: water-soluble albumins, salt-soluble globulins, alcohol-soluble prolamins, and acid- or alkali- soluble glutelins (Osborne and Mendel, 1914). As a monocot, maize embryo contains about 10% proteins, of which 60–80% are storage proteins, especially albumins and globulins (Shewry and Halford, 2002).

In comparison to albumins, water-insoluble globulins in seeds are easily extracted to a relatively high purity using a dilute saline solution. Globulin-1 (GLB1) is the most abundant protein present in maize embryos, followed by the second most abundant protein globulin-2 (GLB2). Both globulins are rapidly degraded in early stages of seed germination to provide a source of nitrogen and carbon for the seedling development (Kriz, 1989). GLB1 and GLB2 are encoded by the single genes *Glb1* and *Glb2*, respectively; whose expressions are found in developing embryo but not in endosperm and other tissues of maize seedlings (Belanger and Kriz, 1989; Kriz and Wallace, 1991).

Maize hybrids are widely cultivated for production as their F_1_ generation has superior qualities than those of parental generation, a phenomenon known as heterosis. Previous studies have provided insights into the mechanism of heterosis on molecular level (Jahnke et al., 2010; Marcon et al., 2010, 2013; Fu et al., 2011; Guo et al., 2013; Hu et al., 2016; Li et al., 2017). For instance, maize hybrid embryos have been reports to have strong crossbreeding advantages at the early stage after fertilization (Wang, 1947; Meyer et al., 2007). Organ- or tissue-specific regulatory mechanisms of heterosis have been revealed by comparing non-additive protein accumulation between rice and maize embryo (Marcon et al., 2010). Fu et al. (2011) observed the important roles of dominance, partial dominance, and over-dominance in regulating seed germination in five elite maize hybrids and their parents. Further, it has been indicated that the changes in accumulation patterns of various proteins between two parental lines and the hybrid can be attributed to the altered pattern of gene expression at the translational level in the hybrid (Guo et al., 2013). However, the comparison of the accumulation patterns of seed storage proteins in maize embryos between hybrids and their parental lines have not been reported.

In the present study, Zheng 58 and Chang 7-2, which are two elite inbred lines in China, and their reciprocal hybrids (Zheng 58 × Chang 7-2 and Chang 7-2 × Zheng 58) were used to explore relative influence of parents on the accumulation patterns of seed storage proteins. For this purpose, seed salt soluble proteins (SSPs) were selected in our study. The SSPs were selectively extracted from maize mature embryos after extensive removal of water-soluble albumin and separated using two-dimensional gel electrophoresis (2-DE), followed by mass spectrometry (MS) analysis. The relatively abundant SSPs and the expression patterns of non-additive proteins in the hybrids were analyzed.

## Materials and methods

### Maize materials

**Figure 1:**
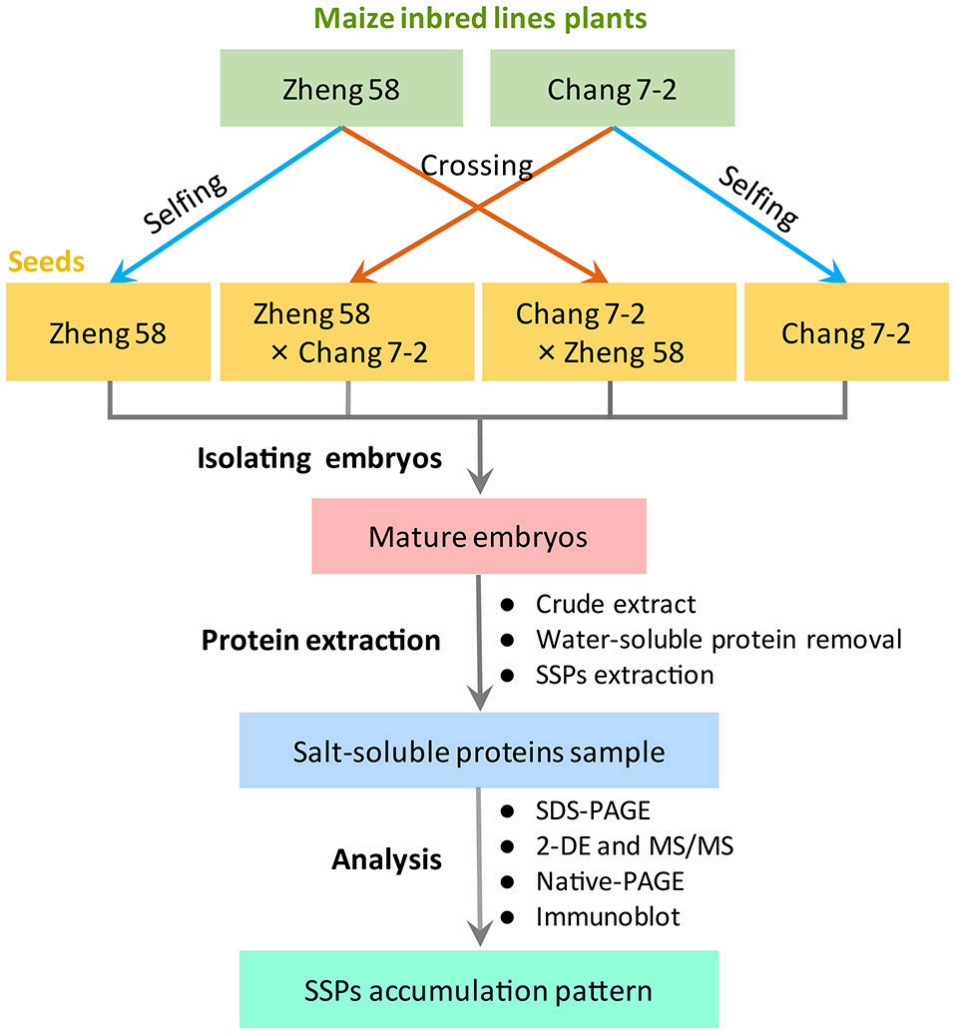
Experimental design in the present study.

Maize inbred lines Zheng 58 and Chang 7-2 were grown in the experimental farm of Henan Agricultural University (Zhengzhou, China; 113°42′ E, 34°48′ N). Thirty plants of each inbred lines were strictly self-pollinated or reciprocal cross-pollinated, involving three independent biological replicates (Figure 1). Mature seeds of Zheng 58, Chang 7-2, Zheng 58 × Chang 7-2, and Chang 7-2 × Zheng 58 were used in this study.

Dry maize seeds were soaked in distilled water overnight to soften starchy endosperm. For each biological replicate, embryos of 10 maize seeds were manually separated and used for protein extraction.

### Antibody production

The antibody for GLB1 (previously designated as vicilin, UniProtKB accession Q03865) was produced as described (Wu et al., 2013). A 17 amino acid sequence (RQSQGGESERERDKGRR) of globulin-1 was chosen and chemically synthesized by a solid phase method using an Fmoc strategy peptide synthesizer (PSSM-8, Shimadzu, Kyoto, Japan). The synthetic 17-mer peptide was purified by high performance liquid chromatography and its sequence was checked by MS. Subsequently, the peptide was coupled with bovine serum albumin (BSA). About 500 μg of the peptide in 0.5 ml of phosphate buffer (pH 7.4) was mixed with 1.0 ml of Freund's complete adjuvant. This mixture was injected into a 4-month old New Zealand white female rabbit to produce polyclonal antibodies. This operation was repeated three times at 20-day intervals using 250 μg of synthetic peptide. After the final booster, the rabbit was bled, and anti-serum was separated by keeping the blood at 4°C overnight. The anti-serum containing polyclonal antibodies was passed through a kromasil C18-5 column and used in the experiment. We designate the antibody as anti-GLB1 in the present study.

### Protein extraction

Maize embryos from three independent biological replicates were separately pulverized with liquid nitrogen using mortar and pestle, and further ground with cold acetone (under native conditions) or cold acetone plus 5 mM DTT (under denaturing conditions). After acetone wash for two times, the tissue powder was air-dried and used for protein extraction. Protein extraction was performed at 4°C unless otherwise stated.

Total protein of maize embryo was extracted as described previously (Wu et al., 2015) with slight modifications. Briefly, the dried powder of embryo tissues (0.2 g) was ground in a buffer containing 0.25 M Tris-HCl (pH 7.5), 1% SDS, 14 mM DTT and a cocktail of protease inhibitors. Next, the homogenate was centrifuged at 12,000 g for 10 min, and the supernatant was subjected to phenol extraction.

SSPs were extracted as described (Cross and Adams, 1983) with certain modifications. The dried powder of embryo tissues (0.2 g) was first extracted with 2 ml distilled water by shaking for 10 min to remove water-soluble albumins. After centrifugation at 12,000 g for 10 min, the supernatant was taken to a new tube. The precipitate was extracted with 1 ml distilled water thrice by shaking for 10 min and centrifuges as above. The supernatants containing albumins from four extractions were individually collected and subjected to phenol extraction as described (Wu et al., 2014). The precipitate after the fourth water-extraction was re-suspended in a dilute saline solution consisting of 0.5 M NaCl, 5 mM EDTA, and 2 mM PMSF (native condition), or 0.5 M NaCl, 0.1 M DTT, 5 mM EDTA, and 2 mM PMSF (denaturing condition) for SSPs extraction. The mixture of precipitate and dilute saline solution was shaken for 1 h and then centrifuged as above. The resulting supernatant contained SSPs and was subjected to phenol extraction.

GLB1 was purified as described (Xiong et al., 2014) with some modifications. The dried powder of embryo tissues (0.2 g) was homogenized in a cold mortar in 1.0 ml of buffer containing 0.25 M Tris-HCl (pH 7.5), 1% SDS, 14 mM DTT, 5 mM EDTA, and 2 mM PMSF. The homogenate was centrifuged and the supernatant was collected in a new tube for chloroform denaturation. Phase separation was achieved by centrifugation as above. The interface containing GLB1 was collected and precipitated with five volumes of acetone for 2 h (−20°C).

The protein pellet was dissolved in an SDS containing buffer for SDS–PAGE, or in a non-reducing, non-denaturing sample buffer for Native PAGE, or in rehydration buffer containing 7 M urea, 2 M thiourea, 2% (w/v) CHAPS, 20 mM DTT, 0.5% (v/v) IPG buffer (pH 4–7, GE Healthcare) for 2-DE. Protein extract was clarified by centrifugation prior to electrophoresis. Protein content was estimated by Bradford microassay (Bio-Rad) with BSA standards.

### Electrophoresis and immunoblot analysis

Native PAGE (8% gel) and SDS-PAGE (4.75% stacking gel and 12.5% resolving gel) were performed according to Laemmli (1970).

For 2-DE, first dimension isoelectric focusing (IEF) separation was performed using 11 cm linear pH 4–7 IPG strips (Bio-Rad, USA). The strips were loaded with equal amount of proteins (600 μg in 220 μl) and passively rehydrated for 12 h at 20°C on PROTEAN IEF CELL system (Bio-Rad, USA). IEF and subsequent SDS-PAGE were performed as previously described (Wu et al., 2015).

After electrophoresis, proteins in the gels were visualized using Coomassie brilliant blue (CBB) G250 or electrophoretically transferred onto polyvinylidene difluoride membrane (Hybond-P, GE healthcare) in a transfer buffer (20% v/v methanol, 50 mM Tris, 40 mM glycine) for 1.5 h. After incubated with 5% skimmed milk in TBST buffer (50 mM Tris-HCl, pH 7.5, 0.15 M NaCl, 0.1% Tween-20) for 2 h, the membrane was incubated with anti-GLB1 polyclonal antibody in TBST buffer (1:3,000 dilution) for 1 h, and subsequently with peroxidase-conjugated goat anti-rabbit IgG (1:2,000 dilution) for 1 h. Finally, the blot was visualized by using 0.1 M Tris-HCl (pH 7.5), 0.08% 3,3′-diaminobenzidine tetrahydrochloride and 0.05% H_2_O_2_.

### Statistical analysis

CBB stained 2DE gels were photographed; and digital images were processed and analyzed using PDQUEST software (Bio-Rad, USA). The volumes of every detected spot were normalized. After normalization, statistical analyses for those spots were carried out by one-way analysis of variance (ANOVA) model. One-way ANOVA was performed based on three biological replications. The spots with at least two-fold changes with statistically significant (*t*-test with a *P* < 0.05) and reproducible changes in abundance among maize hybrids and parental lines were selected for MS analysis. The patterns of non-additive proteins were analyzed according to Hoecker et al. (2008). Statistical analysis for protein contents in maize embryos (five biological replications) was performed by one-way ANOVA.

### Mass spectrometry

The selected protein spots were extracted, digested and analyzed by the MALDI-TOF/TOF analyzer (AB SCIEX TOF/TOF-5800, USA) as described previously (Wu et al., 2015). MALDI-TOF/TOF spectra were acquired in the positive ion mode and automatically submitted to Mascot 2.2 (http://www.matrixscience.com) for identification against NCBInr database (version February 17, 2017; species, *Zea mays*, 279566 sequences). Only significant scores defined by Mascot probability analysis greater than “identity” were considered for assigning protein identity. All of the positive protein identification scores were significant (*P* < 0.05).

### Bioinformatics analysis

BLAST search in the protein database UniProKB (http://www.uniprot.org/) was carried out to search the homologs of uncharacterized maize embryo proteins. The identified proteins were functionally classified according to the annotations in the UniProtKB database. Amino acid composition and grand average of hydropathicity (GRAVY) analyses were performed using ProtParam tool (http://web.expasy.org/protparam/).

## Results

### The extraction of SSPs in maize embryos

**Figure 2:**
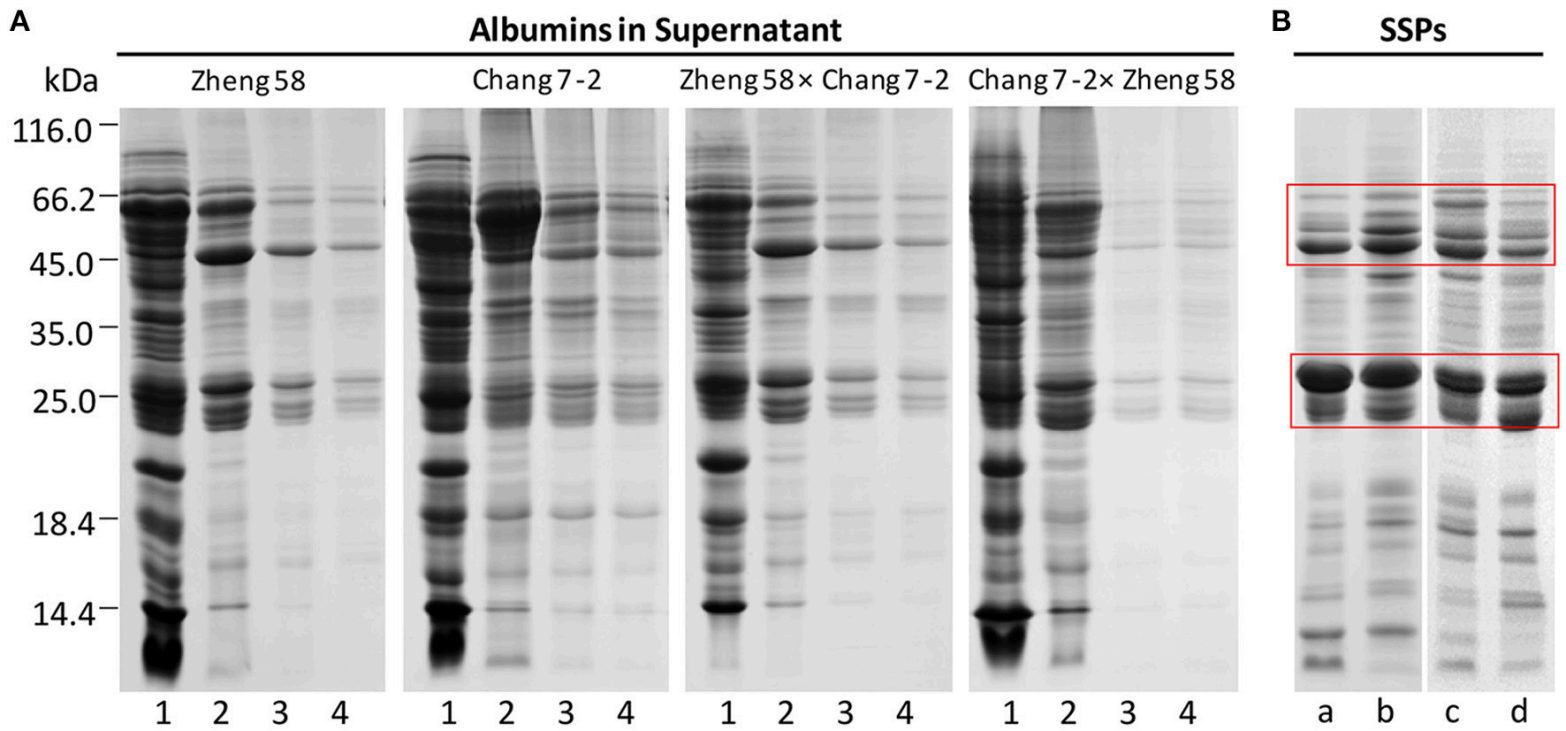
SDS-PAGE of albumins and SSPs in maize embryos. 1–4, representing the times of albumin extraction; a–d, representing Zheng 58, Zheng 58 × Chang 7-2, Chang 7-2, and Chang 7-2 × Zheng 58, respectively; red box, highlighting the regions of enriched SSPs. **(A)** Removal of albumins by water extraction. **(B)** Enrichment of SSPs after depletion of albumins.

To extract SSPs from maize embryos, first, removal of water-soluble albumins is necessary. The residual albumins in maize embryo tissues (precipitates) became less and less with water-extraction times. The removal effect of albumins was evaluated by SDS-PAGE (Figure 2A). Most albumins could be effectively removed in the first three rounds of extraction as their abundance gradually decreases. Next, the SSPs in water-extracted embryo tissues were extracted with 0.5 M NaCl. SDS-PAGE demonstrated that SSPs were greatly enriched with depletion of albumins, mainly in the range of 20–30 and 43–66 kDa (Figure 2B, red boxes).

### Comparison of protein contents in embryos of the reciprocal hybrids and parental inbred lines

Total proteins and SSPs in embryos of each maize genotype were extracted and quantified (Table 1). The total protein content in maize embryo was 31.3–34.2 mg/g dry weight, and the SSPs content was 3.3–4.3 mg/g dry weight. In terms of the total protein and SSPs contents, the hybrid Zheng 58 × Chang 7-2 was similar to Zheng 58, and Chang 7-2 × Zheng 58 was similar to Chang 7-2. In addition, total protein contents in Zheng 58 and Zheng 58 × Chang 7-2 were higher than those of Chang 7-2 and Chang 7-2 × Zheng 58 whereas reverse was the trend in the case of SSPs.

**Table 1:** Average protein contents of total proteins and SSPs in maize embryos.

**Maize seeds**	**Total proteins (mg/g dry weight)**	**SSPs (mg/g dry weight)**	**SSPs (%)**
Zheng 58	33.79 ± 0.76^ac^	3.64 ± 0.01^b^	10.77
Chang 7-2	31.30 ± 1.06^b^	4.31 ± 0.01^a^	13.77
Zheng 58 × Chang 7-2	34.18 ± 0.23^a^	3.49 ± 0.03^b^	10.21
Chang 7-2 × Zheng 58	31.85 ± 0.07^b^	4.21 ± 0.01^ac^	13.19

*Column 2 and 3 in the table represent mean of five independent replications along with standard deviation. Different letters indicate significant differences according to the Tuckey-test algorithm of the students range (α = 0.05). Five biological replicates were studied for each sample and one-way ANOVA was used for data analysis*.

### Comparative proteomic analysis of relatively abundant SSPs in hybrids and parental inbred lines

**Figure 3:**
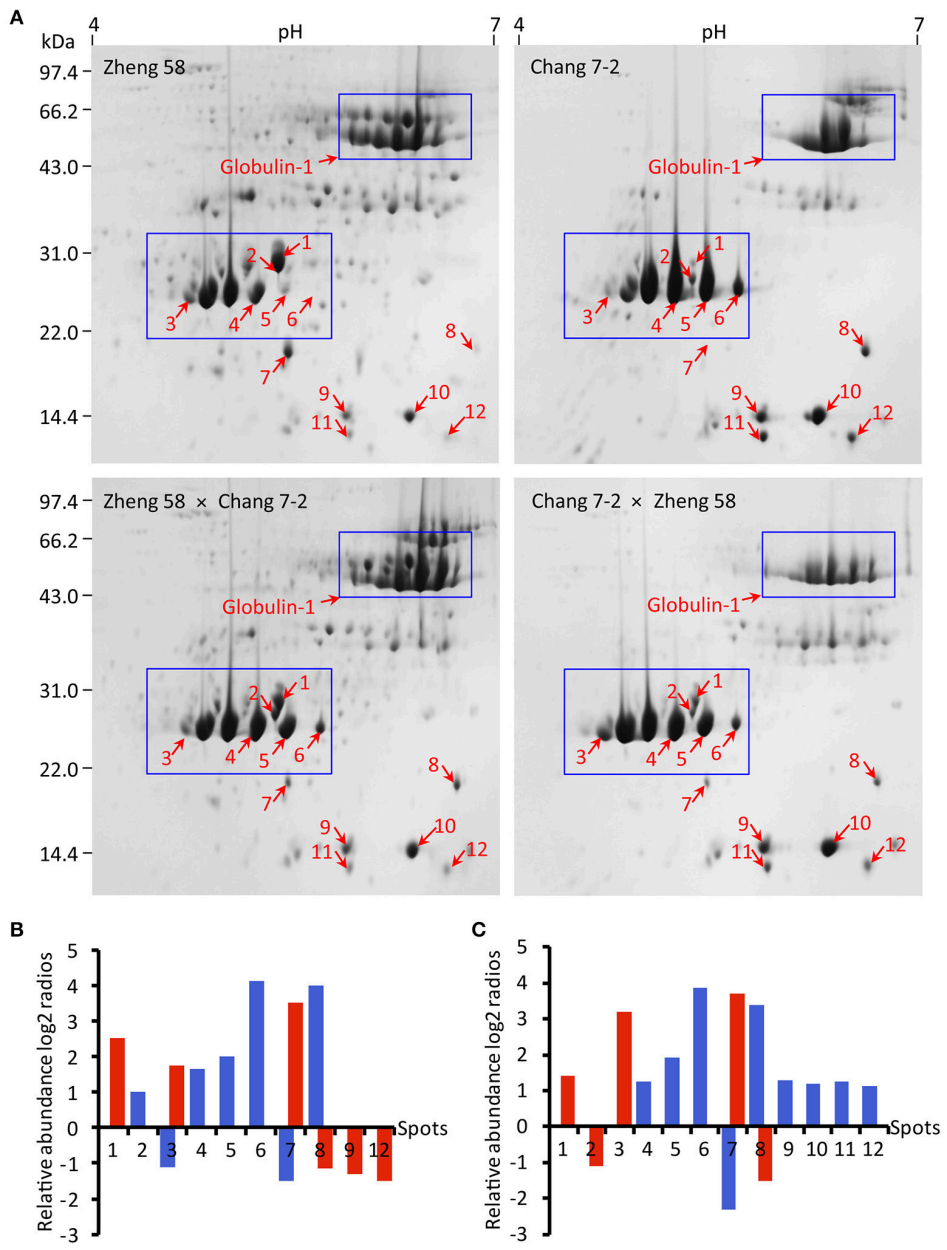
2-DE profiles, abundance, and comparison of SSPs in maize embryos of hybrids and their parents. Maize embryo SSPs (600 μg) were resolved by IEF using 11 cm pH 4–7 IPG dry Strip. Secondary SDS-PAGE was carried out on a 12.5% resolving gel. **(A)** Spots of relatively abundant protein in hybrids and their parents with at least two-folds change in abundance are indicated with red arrows. Blue boxes indicate the regions concentrated with differential SSPs. **(B,C)** Indicating log2 ratios of Zheng 58 × Chang 7-2 and Chang 7-2 × Zheng 58 to their parents blue represents Zheng 58 and red represents Chang 7-2. Log2 ratio > 1 represents more than two-folds of abundance change. Spot volumes were determined using the PDQuest software.

In the preliminary experiment, linear gradient strips of both pH 3–10 and pH 4–7 were used for 2-DE; however, most proteins were resolved in pH 4–7 range strip. Maize embryo SSPs from Zheng 58 × Chang 7-2, Chang 7-2 × Zheng 58, Zheng 58 and Chang 7-2 were further separated by 2-DE with IPG strip pH 4–7. Images of 2-DE were analyzed with PDQuest software (Figure 3A, Figure S1). A spot-to-spot matching between the SSPs profiles of reciprocal hybrids and their parental inbred lines was performed. The gels from Zheng 58 were taken as master gels and approximately 222 ± 10 spots were reproducibly detected in three biological replicates, meanwhile, 183 ± 16 spots in Zheng 58 × Chang 7-2, 39 ± 6 spots in Chang 7-2, and 58 ± 7 spots in Chang 7-2 × Zheng 58 were also reproducibly detected. Subsequently, spot-to-spot matching between the reciprocal hybrids revealed that the SSPs profile of Zheng 58 × Chang 7-2 had higher similarity with that of Zheng 58 than it had with SSPs profile of Chang 7-2. Similarly, SSPs profile of Chang 7-2 × Zheng 58 had more resemblance with that of Chang 7-2 than it had with SSPs profile of Zheng 58. This higher “similarity” or “resemblance” was observed especially in two regions, with molecular ranges of ~35–45 and 60–70 kDa (Figure 3A, blue boxes), both revealing a matroclinous inheritance.

Spot-to-spot comparison revealed 12 spots with at least two-fold change in abundance between the reciprocal hybrids and their parental inbred lines (Figures 3B,C). Among them, seven spots accumulated in higher abundance in the reciprocal hybrids were comparable to those in their parents. We found that four spots (spots 4, 5, 6, and 8) in the reciprocal hybrids were similar to those in Zheng 58 and three spots (spots 1, 3, and 7) in the reciprocal hybrids resembled those in Chang 7-2. Three spots (spots 8, 9, and 12) accumulated in lower abundance in Zheng 58 × Chang 7-2 matched to those in Chang 7-2, and four spots (spots 9–12) accumulated in higher abundance in Chang 7-2 × Zheng 58 compared to those in Zheng 58.

All the spots of relatively abundant proteins were successfully identified by MS/MS, representing five distinct proteins from NCBI or UniProtKB protein databases, including globulin-1-like SSPs (spots 1 and 2), globulin-2 precursor (spot 3), globulin-1 S allele precursor (spots 4, 5, and 6), 16.9 kDa class I heat shock protein (HSP) (spots 7 and 8), and globulin-2 (spots 9–12; Table 2). Most of these proteins were identified in two and more spots (representing different protein isoforms or proteoform) with different p*I* and/or molecular weight in all maize genotypes.

**Table 2:** Identification of relatively abundant SSPs in maize embryos by MS/MS among hybrids and parental inbred lines.

**Spot**	**Protein**	**NCBI accession**	**UniProtKB accession**	**Score**	**Coverage (%)**	**Matched peptide sequences**
1	Globulin-1-like seed storage protein, partial	AQL09051	A0A1D6PIB9	833	75.2	VGGQVVEK; TPPPIIAYNPEEEKGDK; VTSIEEESSEQSSLEVER; IYAIFTSEGINADDPSKPK; GKVTSIEEESSEQSSLEVER; VVAESEAGSVSAVDVADAAGTAYR; LRIYAIFTSEGINADDPSKPK; RVVAESEAGSVSAVDVADAAGTAYR; GDVYNFEQGSILYIQSYPNASR; GFETDVLR; VEAYSSVSNLVK; LGFGVKPEVVEAIK; LHFITMDPGALFLPVQLHADMVFYVHSGR
2	Globulin-1-like seed storage protein, partial	AQL09051	A0A1D6PIB9	768	71.8	LGFGVKPEVVEAIK; TPPPIIAYNPEEEK; TPPPIIAYNPEEEKGDK; VTSIEEESSEQSSLEVER; IYAIFTSEGINADDPSKPK; GKVTSIEEESSEQSSLEVER; VVAESEAGSVSAVDVADAAGTAYR; LRIYAIFTSEGINADDPSKPK; RVVAESEAGSVSAVDVADAAGTAYR; GDVYNFEQGSILYIQSYPNASR; GFETDVLR; VEAYSSVSNLVK; LHFITMDPGALFLPVQLHADMVFYVHSGR
3	Globulin 2 precursor	NP_001295419	Q7M1Z8	519	37.1	VVMLLSPVVSTSGR; FTHELLEDAVGNYR; GQGYFEMACPHVSGGR; AFLQPSHYDADEVMFVK; LLAFGADEEQQVDRVIGAQK; EGDVMVIPAGAVVYSANTHQSEWFR; REEWEK; VAELEAAPR; QSKGEITTASEEQIR; GEITTASEEQIR; FEEFFPIGGESPESFLSVFSDDVIQASFNTR
4	Globulin-1 S allele precursor	ACG48473	B6UGJ0	448	48.1	REEWEK; EGEGVIVLLR; GEITTASEEQIR; GSRGEGGDSGSSSSSK; VVMLLSPVVSTSGR; FTHELLEDAVGNYR; GQGYFEMACPHVSGGR; TFLQPSHYDADEVMFVK; VAELEAAPR; VLERFTHELLEDAVGNYR; LLAFGADEEQQVDRVIGAQK; EGDVMVIPAGAVVYSANTHQSEWFR; KPTHSNSHGRHYEITGDECPHLR; FEEFFPIGGESPESFLSVFSDDVIQASFNTR
5	Globulin-1 S allele precursor	ACG48473	B6UGJ0	450	42.8	TFLQPSHYDADEVMFVK; QSKGEITTASEEQIR; VLERFTHELLEDAVGNYR; FTHELLEDAVGNYR; LLAFGADEEQQVDRVIGAQK; VVMLLSPVVSTSGR; FEEFFPIGGESPESFLSVFSDDVIQASFNTR; EGEGVIVLLR; GEITTASEEQIR; REEWEK; VAELEAAPR; EGDVMVIPAGAVVYSANTHQSEWFR; GQGYFEMACPHVSGGR
6	Globulin-1 S allele precursor	ACG48473	B6UGJ0	449	42.8	GQGYFEMACPHVSGGR; TFLQPSHYDADEVMFVK; FTHELLEDAVGNYR; EGEGVIVLLR; LLAFGADEEQQVDRVIGAQK; EGDVMVIPAGAVVYSANTHQSEWFR; VFEKQSK; REEWEK; VAELEAAPR; QSKGEITTASEEQIR; GEITTASEEQIR; VVMLLSPVVSTSGR; FEEFFPIGGESPESFLSVFSDDVIQASFNTR;
7	16.9 kDa class I heat shock protein 1	ACG36285	B6TGQ2	275	59.9	ADLPGVKK; SSGQFVRR; FRLPENAK; AALENGVLTVTVPKAEVK; AALENGVLTVTVPK; VEVEDGNVLLISGQR; EEVKVEVEDGNVLLISGQR; SSVFDPFSVDLFDPFDSMFR; ETPEAHVFK; RSSVFDPFSVDLFDPFDSMFR
8	16.9 kDa class I heat shock protein 1	ACG31332	B6T2J9	263	70.1	AALENGVLTVTVPK; VEVEDGNVLLISGQR; SIVPPSLSSSAASETAAFASAR; ETPEAHVFK; EEVKVEVEDGNVLLISGQR; ADLPGVKK; SSGQFVRR; FRLPENAK; SSVFDPFSVDLFDPFDSMFR
9	Globulin-2	ONM10183	A0A1D6L7G1	441	37.0	GHGREEEEER; VFLAGTNSALQK; LLAFGADEEQQVDR; EEEEEREEEQGGGGGQK; VFLAGTNSALQKMDRPAK; EGSVIVIPAGHPTALVAGEDK; IREGSVIVIPAGHPTALVAGEDK
10	Globulin-2	ONM10183	A0A1D6L7G1	467	36.1	NLAVLCFEVNASFDDK; EEEEEREEEQGGGGGQK; EGSVIVIPAGHPTALVAGEDK; LLAFGADEEQQVDR; GHGREEEEER; VFLAGTNSALQK; IREGSVIVIPAGHPTALVAGEDK
11	Globulin-2	ONM10183	A0A1D6L7G1	205	31.1	EEEQGGGGGQK; MFVKEGEGVIVLLR; EGSVIVIPAGHPTALVAGEDK; VFLAGTNSALQK; LLAFGADEEQQVDR; IREGSVIVIPAGHPTALVAGEDK
12	Globulin-2	ONM10183	A0A1D6L7G1	371	49.2	MFVKEGEGVIVLLR; LLAFGADEEQQVDR; HYEITGDECPHLR; EEEEEREEEQGGGGGQK; EGSVIVIPAGHPTALVAGEDK; GSMMAPSYNTR; VFLAGTNSALQK; LLDMDVGLANIAR; IREGSVIVIPAGHPTALVAGEDK

*The parameters of Mascot search were as follows: Type of search: combined (MS + MS/MS); Enzyme: trypsin; Fixed modifications: carbamidomethyl (C); Dynamical modifications: oxidation (M); Mass values: monoisotopic; protein mass: unrestricted; Peptide mass tolerance: ±100 ppm; Fragment mass tolerance: ±0.4 Da; Peptide charge state: 1+; Max missed cleavages: 1*.

In this study, two small heat shock proteins (sHSPs) in spots 7 and 8 were found in both hybrids (Table 2). Since the two HSPs remained abundant in embryo pellets even after extensive water-extraction, they should be salt-soluble. These proteins could be regarded as SSPs based on their solubility, because they had a negative GRAVY. Moreover, predicted subcellular locations of HSPs (data not shown) were similar to those of GLBs, though their amino acid compositions was different from that of GLBs (Table 3). Amino acid analysis of GLB1 and GLB2 reveals that both of these globulins contain high amount of arginine and low amounts of cysteine and methionine. The 16.9 kDa HSP1 like GLBs is rich in arginine and glutamate residues (Kriz, 1989; Jaspard and Hunault, 2016); however, HSPs also contain high amounts of lysine.

**Table 3:** Analysis of amino acid composition and grand average of hydropathicity of the identified proteins.

**Protein**	**UniProtKB accession**	**Amino acid composition (%)**																				**GRAVY**
		**A**	**R**	**N**	**D**	**Q**	**E**	**G**	**H**	**I**	**L**	**M**	**F**	**P**	**W**	**S**	**Y**	**T**	**V**	**C**	**K**	
Globulin-1-like seed storage protein, partial (spots 1 and 2)	A0A1D6PIB9	8.0	6.1	2.8	5.2	2.8	10.8	8.0	1.9	5.6	5.6	0.9	3.8	5.6	0.9	8.9	3.8	3.3	10.3	0.0	0.0	−0.416
Globulin 2 precursor (spot 3)	Q7M1Z8	6.7	9.0	2.3	4.4	3.7	10.4	9.7	3.0	3.5	5.8	1.9	4.6	3.9	1.2	10.9	2.1	3.5	7.9	1.2	0.0	−0.625
Globulin-1 S allele precursor (spots 4–6)	B6UGJ0	6.7	9.0	2.3	4.4	3.7	10.4	10.2	3.0	3.5	5.8	1.9	4.6	3.9	1.2	10.4	2.1	3.5	7.9	1.2	4.2	−0.623
Globulin-2 (spots 9–12)	A0A1D6L7G1	9.6	8.5	4.0	4.5	4.0	9.6	11.3	3.4	4.0	6.8	2.8	2.8	4.0	0.0	6.8	2.8	2.8	6.2	1.7	4.5	−0.612
16.9 kDa class I heat shock protein 1 (spots 7 and 8)	ACG31332	5.6	8.9	3.3	5.6	2.2	14.4	6.7	2.2	1.1	6.7	0.0	3.3	4.4	2.2	4.4	0.0	4.4	13.3	0.0	11.1	−0.927

### Non-additive SSPs accumulation patterns in the reciprocal hybrids

Non-additive SSPs accumulation implies means that protein accumulation patterns in hybrids are significantly different from the average value (mid-parent value) of the parental inbred lines (Marcon et al., 2013). Among the 12 spots of relatively abundant proteins, nine spots displayed non-additive accumulation patterns in both hybrids. In Zheng 58 × Chang 7-2, three spots (spots 6, 7, and 8) showed high parent expression (+), two (spot 3 and 5) displayed partial dominance expression (+−), and two (spots 9 and 12) presented low parent expression (−). In Chang 7-2 × Zheng 58, four spots (spots 6, 9, 10, and 11) exhibit high parent expression, three (spot 5, 7, and 8) showed partial dominance expression, and one (spot 3) indicated above high parent expression (++) (Table 4). Among the non-additive SSPs, 7% (1/15) were classified as above high parent expression (+) while 13% (2/15) displayed low parent expression (−). Besides, majority of non-additively expressed proteins (12/15, 80%) revealed a similar abundance with the high parent expression and partial dominance, suggesting that dominance and partial dominance might be the major patterns for the relatively abundant SSPs in both of the hybrids.

**Table 4:** Non-additive SSPs accumulation patterns in the reciprocal hybrids.

**Hybrids**	**Non-additive protein type**			
	**++^a^**	**+^b^**	**+−^c^**	**−^d^**
Zheng 58 × Chang 7-2	0	3	2	2
Chang 7-2 × Zheng 58	1	4	3	0

a ++, above high parent expression;

b +, high parent expression;

c +−, partial dominance;

d *−, low parent expression*.

### Specific accumulation of globulin-1 in the reciprocal hybrids and parental inbred lines

GLB1 is the most abundant seed storage protein in maize embryos. Thus, we produced its antibody anti-GLB1 to further analyze its accumulation in the reciprocal hybrids and parental inbred lines. Generally, it is sufficient to elicit an effective immunization response by using a 10–20 amino acid peptide (Wu et al., 2013; Lee et al., 2016). The immunoreactivity of the polyclonal antibody anti-GLB1 was examined using SDS-PAGE, 2-DE, and immunoblot analyses (Figure S2). The immunoblot analysis ascertained anti-GLB1 was highly specific for 60–70 kDa proteins with a strong reactivity. 2-DE blot analysis also confirmed the specificity of the antibody for the detection of GLB1 proteoforms (Figure 3, Figure S2), which were identified by MS/MS in our previous study (Xiong et al., 2014).

**Figure 4:**
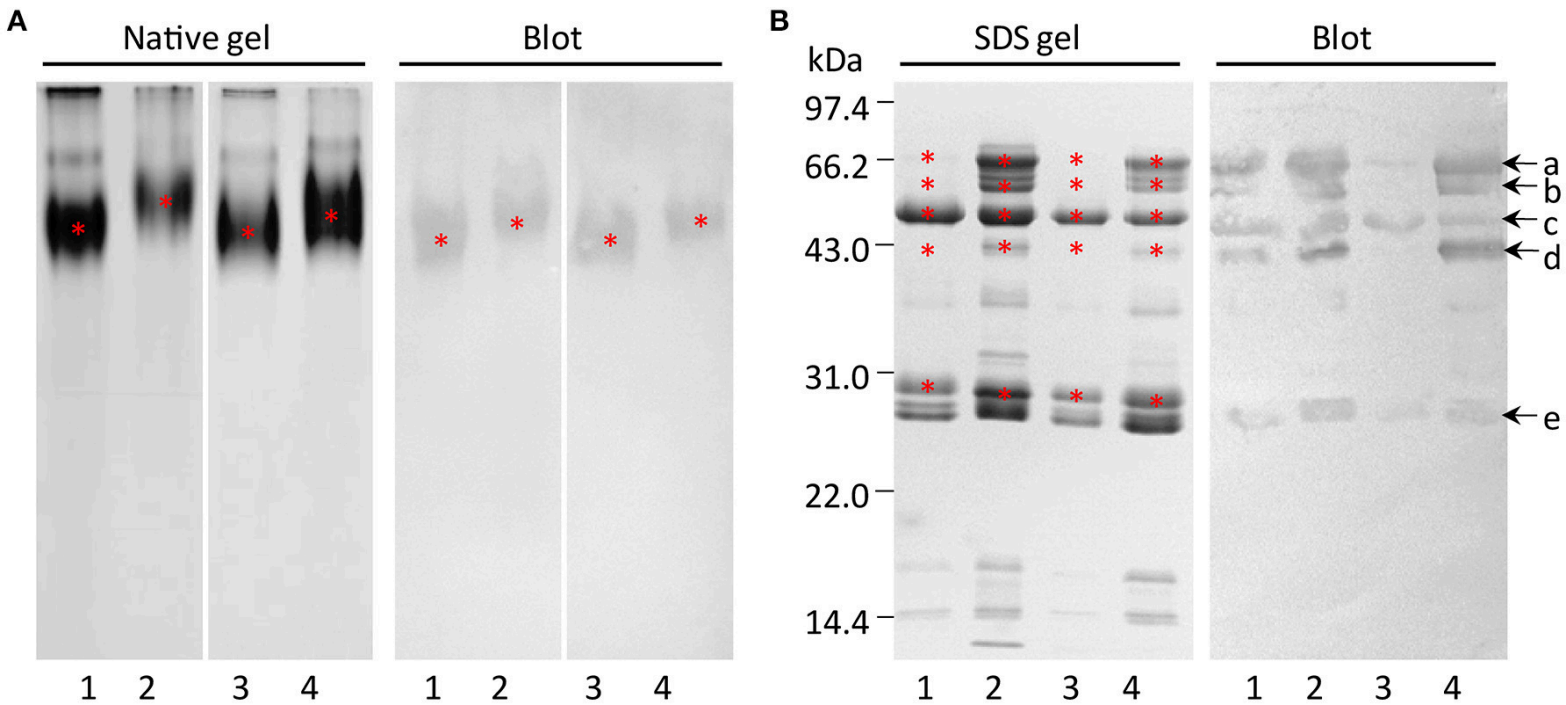
Subunit compositions of globulin-1 in maize embryos of the hybrids and parental inbred lines. **(A)** Asterisks indicate natural form of globulin-1. **(B)** Native globulin-1 was extracted and excised from the gel **(A)**, resolved under non-reducing conditions or reducing conditions, and immunodetected with anti-globulin-1 polyclonal antibody (1:3,000 dilution). Asterisks or arrows indicate the subunits of globulin-1. 1–4, representing Zheng 58, Chang 7-2, Zheng 58 × Chang 7-2, and Chang 7-2 × Zheng 58, respectively.

In order to investigate the subunits of GLB1 in maize embryos, the SSPs were first subjected to Native-PAGE, and a natural form of GLB1 with high molecular weight was detected by immunoblot analysis (Figure 4A). SDS-PAGE showed that band patterns of Zheng 58 × Chang 7-2 had higher similarity with those of Zheng 58 than they had with Chang 7-2. Similarly, band patterns of Chang 7-2 × Zheng 58 exhibited higher resemblance with those of Chang 7-2 than they had with Zheng 58 (Figure 4B). The GLB1 profile of maize hybrids explicitly demonstrated higher resemblance with that of their maternal parent than it did with the profile of paternal parent. This trend was consistent with the above 2-DE results. Immunoblot analysis detected at least five subunits of GLB1 in all the four maize types, with molecular weights of ~71, 63, 50, 43, and 29 kDa (Figure 4B). In addition, similar observations were made when the natural form of GLB1 was analyzed under denaturing and non-reducing conditions, indicating that the subunits of GLB1 might not be linked with disulfide bond.

## Discussion

### Analysis of non-additive SSPs accumulation patterns

Heterosis has been widely utilized in agriculture owing to the superior qualities of F_1_ hybrids than those of their parents. In the present study, SSPs from two maize inbred lines and their reciprocal hybrids were extracted and analyzed using 2-DE to study the accumulation patterns of seed storage proteins. As a result, the 2-DE profiles revealed a matroclinal phenomenon between the reciprocal hybrids and parental inbred lines, although the 2-DE profiles of all four maize types were quite similar.

In the present study, nine SSPs spots among 12 spots of relatively abundant proteins displayed non-additive expression patterns in both hybrids Zheng 58 × Chang 7-2 and Chang 7-2 × Zheng 58, with a consistent change in two biological replicates of all genotypes. Among the non-additively expressed proteins, the high parent expression pattern was the key observation in both of the hybrids, followed by the partial dominant expression. Previous comparative proteomics analyses have demonstrated that non-additive protein accumulation patterns contribute to the performance of heterosis in rice embryos (Wang et al., 2008), maize embryos (Jahnke et al., 2010; Marcon et al., 2010), and maize roots (Hoecker et al., 2008; Marcon et al., 2013). Therefore, the SSPs expression pattern between maize hybrids and their parental inbred lines in our study was consistent with that in previous studies.

The expression of proteins in hybrids is always influenced by genetic variations and environmental effects. In fact, maize embryo protein profiles in the present study were genetically controlled by both parents. However, environmental effect on SSPs accumulation was negligible because all maize plants were cultivated under same conditions. Thus, the differences in SSPs accumulation between Zheng 58 × Chang 7-2 and Chang 7-2 × Zheng 58 were mainly because of the maternal plants. In addition, the accumulation of several SSPs differed between two hybrid combinations. For example, GLB2 (spot 9) was identified as low parent expression in Zheng 58 × Chang 7-2, but shown to be high parent expression in the Chang 7-2 × Zheng 58, indicating the complexity in heredity of the SSPs.

### Relatively abundant SSPs in the reciprocal hybrids and parental inbred lines

In our study, the identified 12 relatively abundant SSPs spots were classified into three major categories GLB1, GLB2, and HSPs. Most of these proteins exist in at least two proteoforms. Maize GLB1 in our study was also found to consist of several major proteoforms with similar sizes but different pIs, and this finding was consistent with previous studies (Fasoli et al., 2009; Xiong et al., 2014). Similarly, Marcon et al. (2010) reported that GLB2 in maize embryos was represented by 11 different spots. GLB1 and GLB2 are encoded by the single-copy *Glb1* and *Glb2* genes, respectively (Schwartz, 1979; Kriz, 1989). Both the genes are highly polymorphic with several different alleles (Belanger and Kriz, 1989; Kriz and Wallace, 1991). Thus, the diverse proteoforms of GLBs have originated from the gene polymorphism, protein modifications or degradation.

A previous study reported that GLB1 is a multi-subunit protein and contained four subunits of 63, 45, 26, and 23 kDa (Kriz, 1989). However, our data suggest that it included at least five subunits of ~71, 63, 50, 43, and 29 kDa in all four genotypes.

HSP70 displayed non-additive accumulation in rice embryos (Wang et al., 2008), 3.5-day-old maize roots (Hoecker et al., 2008), and developing maize embryos (Marcon et al., 2013). Similarly, we found here that HSP16.9 showed high parent expression in Zheng 58 × Chang 7-2. HSPs are similar to GLBs in the terms of amino acid residues (Kriz, 1989; Jaspard and Hunault, 2016). GLBs are commonly named on the basis of solubility whereas sHSPs are named by their molecular masses and functions (Bakthisaran et al., 2015). A previous research suggested that HSP20 might be associated with the appearance of heterosis during germination (Ouyang et al., 2009).

In summary, the SSPs in maize are important in determining the processing and nutritional qualities of maize seeds. The present study provides a high resolution quantitative comparison of proteome complexity of reciprocal hybrids and their parental inbred lines in maize embryos. The results suggest that the expression pattern of SSPs in maize hybrids exhibits higher resemblance with that of maternal plant than it does with that of paternal plant. However, the expression pattern of non-additively accumulated proteins is different in the reciprocal hybrids which may be because of protein interaction and post-translational modification. With the rapid development of modern molecular biology technology, research on crop SSPs will facilitate further improvements in the quality of crops through genetic engineering.

## Author contributions

WW: Conceiving the study. FN, HZ, and LJN: Conducting the experiments. FN, WW, and XLW: Analyzing the data. FN and WW: Writing the manuscript. XLW, ZKW, HY, and WW: Revising the manuscript.

### Conflict of interest statement

The authors declare that the research was conducted in the absence of any commercial or financial relationships that could be construed as a potential conflict of interest.

## Supplementary material

Figure S1 2-DE profiles of SSPs in maize embryos of hybrids and their parents. Maize embryo SSPs (600 μg) were resolved by IEF using 11 cm pH 4–7 IPG dry Strip. Secondary SDS-PAGE was carried out on a 12.5% resolving gel. Spots of relatively abundant protein in hybrids and their parents with at least two-folds change in abundance are indicated with red arrows.

Figure S2 Specificity analysis of the antibody anti-GLB1 by 2-DE immunoblotting. Enriched GLB1 (500 μg) from maize embryo were resolved by IEF using 11 cm pH 4–7 IPG dry Strip. Secondary SDS-PAGE was carried out on a 12.5% gel. Corresponding region of the red box marked was used for blot analysis. Blot probed with anti-GLB1 (1:3,000 dilution).
