# Supplementary figures and images for: Accumulation Profiles of Embryonic Salt-Soluble Proteins in Maize Hybrids and Parental Lines Indicate Matroclinous Inheritance: A Proteomic Analysis
Source: Front Plant Sci. 2017 Oct 25;8:1824. doi: 10.3389/fpls.2017.01824 (PMC5661082; doi:10.3389/fpls.2017.01824)

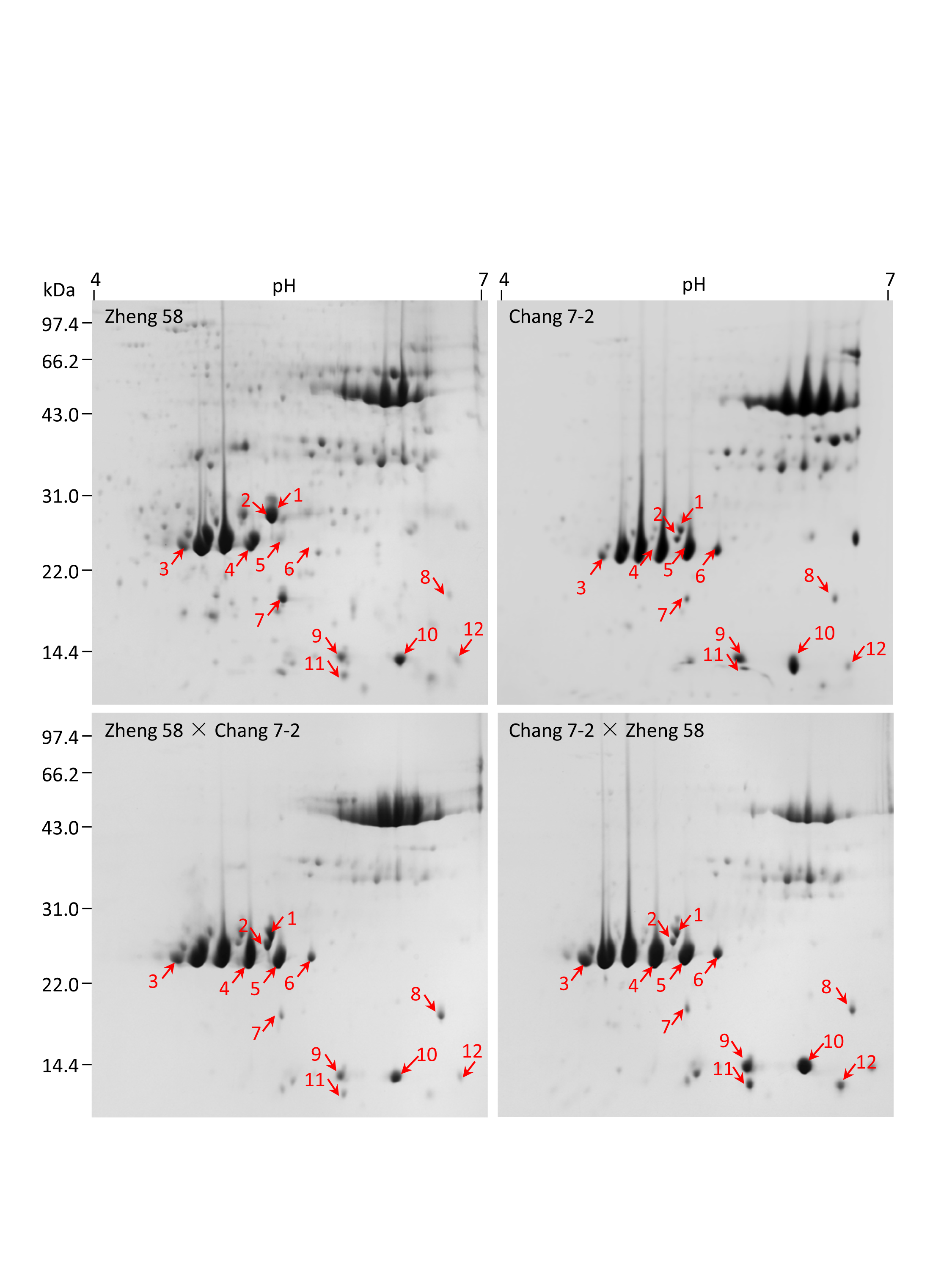

Supplement: Figure S1 — 2-DE profiles of SSPs in maize embryos of hybrids and their parents. Maize embryo SSPs (600 μg) were resolved by IEF using 11 cm pH 4–7 IPG dry Strip. Secondary SDS-PAGE was carried out on a 12.5% resolving gel. Spots of relatively abundant protein in hybrids and their parents with at least two-folds change in abundance are indicated with red arrows. [file Image1.TIF]

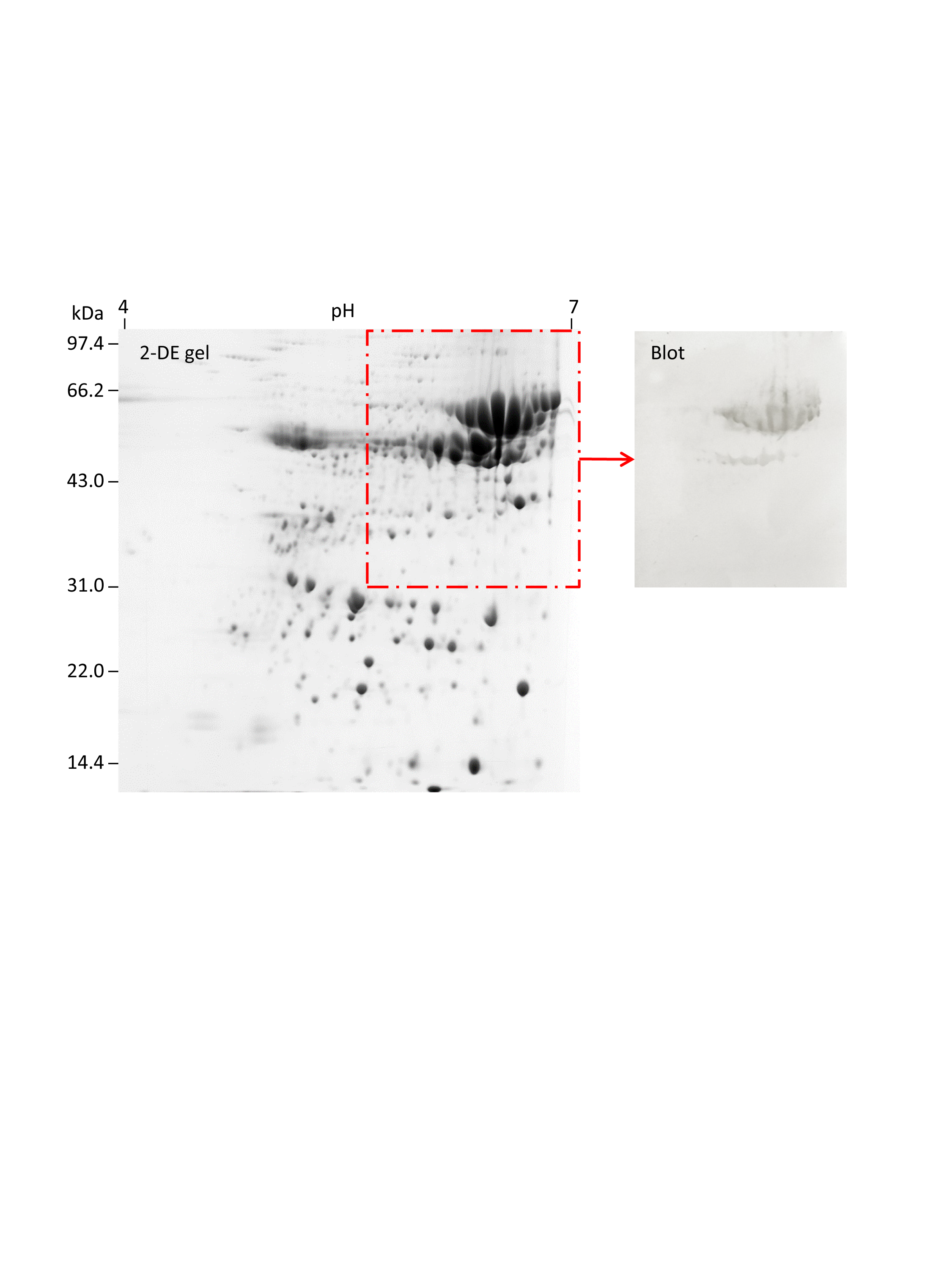

Supplement: Figure S2 — Specificity analysis of the antibody anti-GLB1 by 2-DE immunoblotting. Enriched GLB1 (500 μg) from maize embryo were resolved by IEF using 11 cm pH 4–7 IPG dry Strip. Secondary SDS-PAGE was carried out on a 12.5% gel. Corresponding region of the red box marked was used for blot analysis. Blot probed with anti-GLB1 (1:3,000 dilution). [file Image2.TIF]
